# Symptom Clusters in Patients With Advanced Cancer: A Prospective Longitudinal Cohort Study to Examine Their Stability and Prognostic Significance

**DOI:** 10.1093/oncolo/oyad211

**Published:** 2023-08-03

**Authors:** Diana Simão, Pedro C Barata, Marta Alves, Ana L Papoila, Sónia Oliveira, Peter Lawlor

**Affiliations:** Medical Oncology, Centro Hospitalar Universitário de Lisboa Central, Lisbon, Portugal; Medical Oncology, Centro Hospitalar Universitário de Lisboa Central, Lisbon, Portugal; Division of Solid Tumor Oncology, University Hospitals Seidman Cancer Center, Cleveland, OH, USA; Epidemiology and Statistics Unit, Centro Hospitalar Universitário de Lisboa Central, Lisbon, Portugal; Epidemiology and Statistics Unit, Centro Hospitalar Universitário de Lisboa Central, Lisbon, Portugal; Medical Oncology, Centro Hospitalar Universitário de Lisboa Central, Lisbon, Portugal; Bruyere Continuing Care, Division of Palliative Care, Department of Medicine, Bruyere and Ottawa Hospital Research Institutes, University of Ottawa, Ottawa, Canada

**Keywords:** advanced cancer, palliative care, survival, longitudinal stability, symptom clusters

## Abstract

This study’s purpose was to assess symptom cluster (SC) stability during disease progression and determine their strength of association with survival in patients with advanced cancer . Consecutively eligible patients with advanced cancer not receiving cancer-specific treatment and referred to a Tertiary Palliative Care Clinic were enrolled in a prospective cohort study. At first consultation (D_0_) and in subsequent consultations at day 15 (D_15_) and day 30 (D_30_), patients rated 9 symptoms through the Edmonton Symptom Assessment System scale (0-10) and 10 others using a Likert scale (1-5). Principal components factor analysis with varimax rotation was used to determine SCs at each consultation. Of 318 patients with advanced cancer, 301 met eligibility criteria with a median age of 69 years (range 37-94). Three SCs were identified: neuro-psycho-metabolic (NPM), gastrointestinal, and sleep impairment, with some variations in their constitution over time. Exploratory factor analysis accounted for 40% of variance of observed variables in all SCs. Shorter median survival was observed continuously for NPM cluster (D_0_ 23 vs. 58 days, *P* < .001; D_15_ 41 vs. 104 days, *P*=.004; D_30_ 46 vs. 114 days, *P* = .002), although the presence of 2 or more SCs on D_0_ and D_15_ also had prognostic significance (D_0_: 21 vs. 45 days, *P* = .005; D_30_: 50 vs. 96 days, *P* = .040). In a multivariable model, NPM cluster (D_0_ hazard ratio estimate: HR 1.64; 95%CI, 1.17-2.31; *P* = .005; D_15_ HR: 2.51; 95%CI, 1.25-5.05; *P* = .009; D_30_ HR: 3.9; 95%CI, 1.54-9.86; *P* = .004) and hospitalization (D_0_ HR: 2.27; 95%CI, 1.47-3.51; *P* < .001; D_15_ HR: 2.43; 95%CI, 1.18-5.01; *P* = .016; D_30_ HR: 3.41; 95%CI, 1.35-8.62; *P* = .009) were independently and significantly associated with worse survival. Three clinically relevant SCs were identified, and their constitution had small variations, maintaining a stable set of nuclear symptoms through disease progression. Presence of the NPM cluster and hospitalization maintained their prognostic value over time.

## Teaser

Symptom clusters have been defined as the co-occurrence of two or more symptoms that are interrelated and relatively independent of other symptoms, possibly indicating a common etiology or underlying mechanism. This article reports the results of a study that assessed symptom cluster stability during disease progression to determine their strength of association with survival in patients with advanced cancer.

**Implications for Practice**

Given that symptom clusters (SCs) may have a prognostic value, the importance of regular assessment of symptom burden and SCs in clinical practice should not be underestimated. Despite the need for prospective studies to evaluate the influence of SCs on therapeutic interventions, identification of SCs could provide useful information to guide clinical decisions.

## Introduction

Patients with cancer often experience multiple symptoms either due to the disease itself, its treatment, or other comorbidities, which may increase distress and functional impairment and decrease both quality of life and sense of well-being.^1,2^ Effective symptom control is a major concern throughout cancer treatment; therefore, clinical studies in symptom research have focused predominantly on the treatment of individual symptoms. However, patients with cancer rarely present with a single symptom, which may explain why treating one symptom may have limited benefit on quality of life.^3^

Given the increasing evidence that symptoms in patients with cancer can be interrelated,^4^ research studies have begun to examine the complex relationship between multiple co-occurring symptoms in order to develop effective interventions and improve patients’ outcomes.^3^ The process of comprehensive symptom assessment and management has, in addition to treating a single symptom, adopted a focus on identification of symptom clusters (SCs).^5-7^ Currently known as a dynamic concept, SCs have been defined as the co-occurrence of two or more symptoms that are interrelated and relatively independent of other SCs, possibly indicating a common etiology or underlying mechanism.^4,5^

Investigation of SCs has been performed not only in patients with cancer but also in other chronic diseases.^8^ Regarding patients with cancer, research studies have advanced the identification of SCs mostly in early-stage disease,^9^ with only a few studies published in advanced stage disease.^10,11^ Although the definition of SCs has evolved, consensus is lacking on consistency of symptoms’ dimensions within SCs across different cancer populations^12^ and SCs’ stability or change over time.^13,14^ Although the survival impact of symptom burden has been reported,^15,16^ little is known about the actual prognostic significance of SCs, specifically in relation to patients with advanced cancer. Furthermore, the prospective evaluation of SCs in patients with advanced cancer has been rarely performed longitudinally. There is a need to better understand how SCs evolve over time and potentially affect patient outcomes, so as to develop potential therapeutic interventions to target SCs and optimize symptom management.

We previously reported 3 clinically meaningful SCs at baseline (D_0_) evaluation in patients with advanced cancer.^17^ The presence of more than one cluster as well as the neuro-psycho-metabolic (NPM) cluster was associated with a statistically significant reduction in survival in our cohort.^17^ In this study, we aimed to assess the longitudinal stability of SCs during cancer progression and determine their strength of association with survival.

## Methods

### Study Setting

This study was conducted at Hospital Santo António dos Capuchos (HSAC), a tertiary hospital within the Central Lisbon University Hospital Center, Lisbon, Portugal. The palliative care program at HSAC delivers a daily, weekday outpatient clinic, and same-day assessments for urgent cases. Consecutive referrals to the Palliative Care team were screened for study eligibility.

### Subjects and Eligibility

A prospective cohort study was conducted in patients with advanced cancer referred to a palliative care program between October 2012 and May 2015. Patients were selected for study entry based on the following inclusion criteria: (1) aged 18 years or older, (2) radiological evidence of progressive advanced solid cancer, (3) absence of anticancer therapy at referral, (4) no evidence of dementia or delirium on assessment with the Portuguese versions of the Short Portable Mental Status Questionnaire (SPMSQ)^18^ and the Confusion Assessment Method (CAM)^19^, and (5) ability to provide verbal or written answers to assessment measures and sign written consent. Patients with hematological malignancies were excluded. All patients were enrolled and assessed at first consultation with the palliative care service. This study was approved by the hospital research ethics committee, and informed consent of patients was obtained. Results from the baseline evaluation were published on 2016.^17^ The current study reports cross-sectional data from assessments at 15 and 30 days after the initial baseline consultation. This analysis at 3 time points (baseline, day 15, and day 20) was preplanned and has not been published previously.

### Study Measures and Data Collection

Patient demographics were collected at initial palliative care consultation. Data on patients’ primary cancer location, metastatic sites, and number, type, and date of last active cancer-specific treatment (chemotherapy, radiotherapy, or other cancer-specific therapies, such as tyrosine kinase inhibitors or endocrine therapies) were also gathered. Functional performance status (PS) was assessed with the Eastern Cooperative Oncology Group (ECOG) scale. Cognitive status was evaluated with the Portuguese version of SPMSQ.^18^ The validated Portuguese version of the CAM was used to screen for delirium.^19^ Subjects meeting the study eligibility criteria had a comprehensive assessment of their symptom profile.

A total of 19 symptoms were assessed: patients were requested to routinely complete the revised version of the Edmonton Symptom Assessment Scale (ESAS) and 10 other symptoms using a Likert scale (1-5) on initial and subsequent consultations. The ESAS is a standardized 0-10 numerical rating tool (0 = not a problem, 10 = worst imaginable level of symptom) that is commonly used by palliative care teams to evaluate the intensity of 9 symptoms: pain, dyspnea, lack of appetite, nausea, fatigue, drowsiness, anxiety, depression, and general well-being.^20,21^ It has been revised to facilitate its use (ESAS-r),^22^ and this version of the instrument has been translated into Portuguese.^23^ The ESAS-r was supplemented by questions about the following symptoms: dry mouth, vomiting, constipation, hiccups, sweating, weight loss, dysphagia, sleep disturbance, insomnia (defined as having problems falling asleep), and lack of memory. The intensity of these latter 10 symptoms was scored on a Likert scale: not at all = 1, a little = 2, moderate = 3, severe = 4, and extremely stressing = 5. These symptom evaluations were repeated at 15 (D_15_) and 30 (D_30_) days. Scores rated >2 on ESAS-r or >1 on Likert scale were considered clinically significant and, therefore, reflective of symptom prevalence.

Our electronic records system was used to obtain laboratory data and supplement data collection forms.

### Statistical Analysis

Baseline demographics, disease characteristics, and ESAS-r and Likert scores were summarized with frequencies (percentages) and with mean (SD) or with median and interquartile range (IQR: P_25_-P_75_) as appropriate. The scores from both scales were standardized to *z* scores. Categorical data were analyzed using the Chi-Square test. For each subsequent assessment (15 and 30 days), a principal component factor analysis with varimax rotation was conducted on the standardized clinically significant intensity scores of the 19 symptoms to identify the SCs. The Kaiser-Meyer-Oklin measure was calculated to assess sampling adequacy (scores >0.60 indicate adequate sample size for the analysis). A factor loading >0.40 was considered as an inclusion criterion for each symptom. Derived factors were interpreted and discussed among the research team, and final factor structure included factors deemed to be clinically as well as statistically relevant. Cronbach’s alpha coefficient was used to measure the internal consistency and reliability of the derived SC.

Survival (time to event) analysis was based on the time elapsed between the date of study inclusion and the date of death or last follow-up. At D_15_ and D_30_, Kaplan-Meier analyses with log-rank testing were used to assess group survival estimates. Univariable and multivariable Cox regression models were used to assess the association of demographic and clinical characteristics as well as SCs with time to death at 3 time points (D_0_, D_15_, and D_30_). Among those study variables that are potentially associated with survival time, new variables characterizing each patient as having or not having a SC (for each SC) were considered. Effectively, a prevalence rate of at least 75% for each symptom within the SC was a set requirement for designating the presence of that given SC; otherwise, it was considered absent.

The level of significance *α* = .05 was considered. Data were analyzed using the Statistical Package for the Social Science for Windows (IBM Corp. Released 2013. IBM SPSS Statistics for Windows, Version 22.0. Armonk, NY: IBM Corp.).

## Results

**Figure 1.**
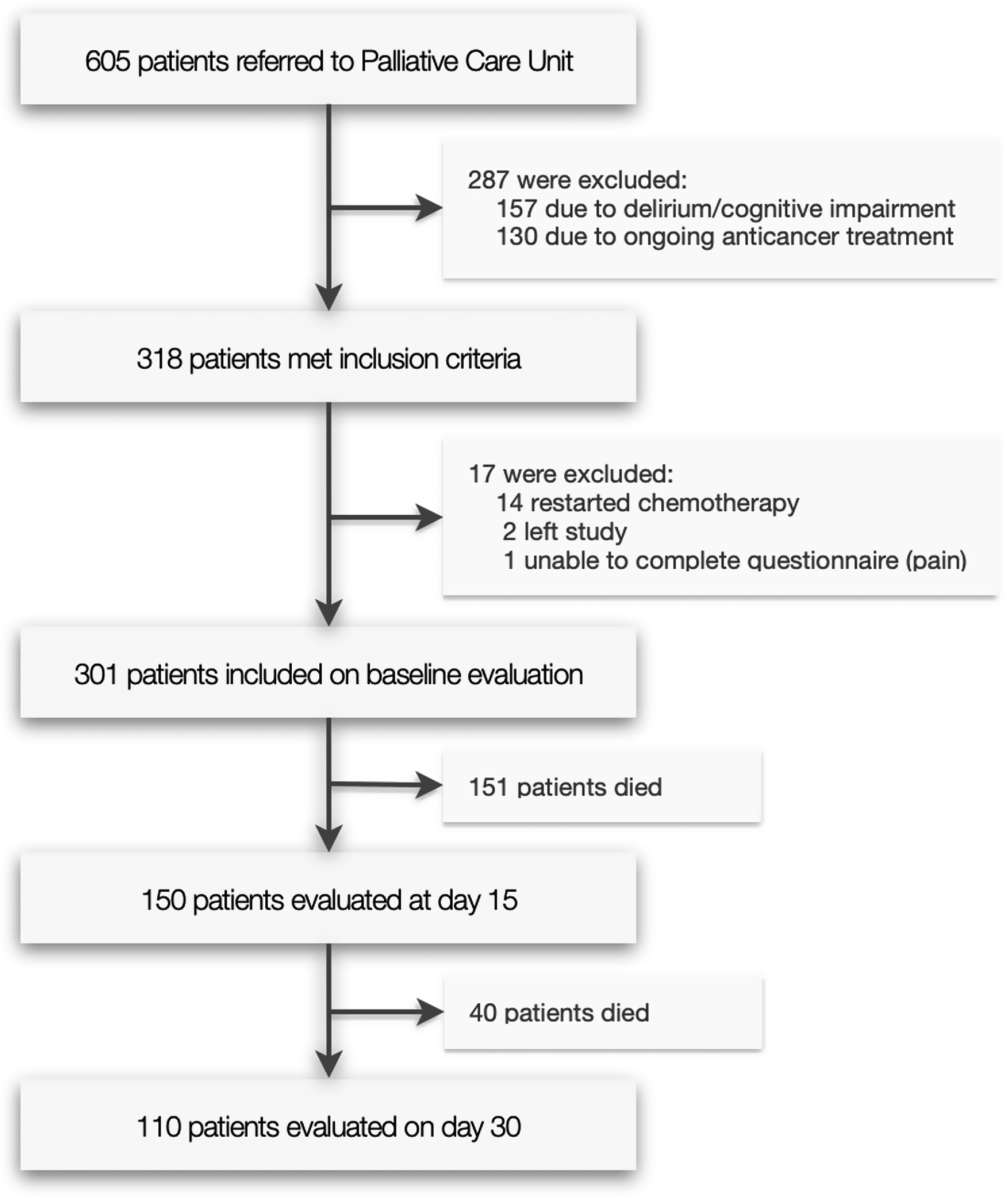
Study participant flow diagram.

Of 605 patients screened for study eligibility, 157 were excluded due to delirium or cognitive impairment, and 130 were on active cancer treatment. Analyses were conducted on the initial symptom assessments from 318 patients, and of these, a further 17 were excluded: 14 restarted cancer treatment, 2 left the study, and one did not complete the questionnaire. Three hundred and one patients were included at baseline, 150 patients at D_15_ evaluation, and 110 patients at D_30_ evaluation (Figure 1). Demographic and clinical details of our study sample at baseline are summarized in Table 1. The median age was 69 years (range: 37-94 years), 172 (57.1%) were male. At baseline assessment, the majority (64.8%) of patients were hospitalized, while in subsequent evaluations most were assessed in an outpatient setting (60.3% on D_15_ and 76.4% on D_30_). The most common primary cancer sites were gastrointestinal (27.6%), lung (17.6%), breast (15.6%), and genitourinary (13.0%). Most patients (95.6%) had metastatic disease, and 64.8% had a PS rating of 3 or 4.

**Table 1.** Baseline clinical characteristics.

Clinical characteristics	Number (%) of patients
	Day 0 (*n* = 301)
Gender	
Male	172 (57.1)
Female	129 (42.9)
Age	
Median (years)	69
Range	37-94
ECOG	
0	3 (1.0)
1	15 (5.0)
2	88 (29.2)
3	142 (47.2)
4	53 (17.6)
Hospitals status	
Outpatient	106 (35.2)
Inpatient	195 (64.8)
Primary cancer site	
Biliary tract	12 (4.0)
Breast	47 (15.6)
Gastrointestinal	83 (27.6)
Genitourinary	39 (13.0)
Gynecological	13 (4.3)
Hepatocarcinoma	14 (4.7)
Lung	53 (17.6)
Pancreas	21 (7.0)
Unknown	9 (3.0)
Other^a^	10 (3.3)
Number of metastatic sites	
0	13 (4.3)
1	37 (12.3)
>1	251 (83.3)

^a^This group includes central nervous system, head and neck, skin, mesothelioma, and sarcomas.

Abbreviation: ECOG: Eastern Cooperative Oncology Group.

Symptom prevalence and intensity during the 3 assessments are summarized in Table 2. The most prevalent symptoms at baseline were tiredness (100%), pain (83.3%), somnolence and dry mouth (82.7%), lack of well-being (77.8%), lack of appetite, and depression (74.8%). At D_15_ and D_30,_ after inclusion in the palliative care program, symptoms were less prevalent and intense (eg, pain prevalence from 83.3% to 45.5%, median intensity score from 6 to 2).

**Table 2.** Symptoms prevalence and intensity over time.

Symptom	Number (%) of patients and symptom intensity scores								
	Baseline (*n* = 301)			Day 15 (*n* = 150)			Day 30 (*n* = 110)		
	Symptom prevalence (%)	Mean score (SD)	Median score (P25-P75)	Symptom prevalence (%)	Mean score (SD)	Median score (P25-P75)	Symptom prevalence (%)	Mean score (SD)	Median score (P25-P75)
ESAS-r									
Pain	251 (83.3)	5.8 (2.2)	6 (4-8)	75 (49.7)	3.0 (3.0)	2 (0-5)	50 (45.5)	2.8 (2.9)	2 (0-5)
Tiredness	301 (100)	6.2 (2.9)	7 (4-9)	127 (84.1)	5.9 (2.9)	6 (4-8)	96 (87.3)	5.8 (2.8)	6 (4-8)
Somnolence	249 (82.7)	4.6 (3.2)	5 (2-7)	89 (58.9)	3.7 (3.2)	4 (0-7)	61 (55.5)	3.6 (3.1)	3 (0-6)
Nausea	91 (30.2)	5.9 (2.2)	7 (4-8)	41 (27.2)	1.6 (2.5)	0 (0-3)	18 (16.4)	1.1 (2.2)	0 (0-2)
Lack of appetite	225 (74.8)	7.0 (2.2)	7 (5-9)	102 (67.5)	4.8 (3.6)	5 (1-8)	70 (63.6)	4.3 (3.3)	4 (1-7)
Dyspnea	91 (30.2)	7.0 (2.3)	7 (5-8)	34 (22.5)	1.4 (2.6)	0 (0-2)	14 (12.7)	1 (2.1)	0 (0-1)
Depression	225 (74.8)	7.0 (2.1)	7 (5-8)	105 (69.5)	4.7 (3.2)	5 (2-7)	69 (62.7)	4 (3.3)	4 (1-7)
Anxiety	178 (59.1)	6.3 (2.2)	7 (4-8)	87 (57.6)	3.57 (3)	3 (0-6)	57 (51.8)	2.9 (2.8)	3 (0-5)
Lack of well-being	234 (77.8)	6.3 (2.0)	7 (5-8)	104 (68.9)	4.57 (2.9)	5 (2-7)	74 (67.3)	4.2 (3)	4 (2-7)
Liker scale									
Vomiting	75 (24.9)	2.8 (0.9)	3 (2-3)	29 (19.2)	1.3 (0.7)	1 (1-1)	16 (14.5)	1.3 (0.7)	1 (1-1)
Constipation	160 (53.2)	3.2 (1.0)	3 (2-4)	70 (46.4)	1.9 (1.2)	1 (1-3)	46 (41.8)	1.8 (1.1)	1 (1-2)
Weight loss	243 (80.7)	3.5 (0.9)	4 (3-4)	104 (68.9)	2.3 (1.3)	2 (1-3)	60 (54.5)	2 (1.2)	2 (1-3)
Dysphagia	89 (29.6)	3.1 (1.1)	3 (2-4)	49 (32.5)	1.6 (0.9)	1 (1-2)	30 (27.3)	1.5 (0.9)	1 (1-2)
Dry mouth	249 (82.7)	3.4 (1.0)	4 (2-4)	110 (72.8)	2.6 (1.3)	2 (1-4)	77 (70)	2.3 (1.2)	2 (1-3)
Sweating	63 (20.9)	2.8 (1.0)	3 (2-3)	50 (33.1)	1.6 (0.9)	1 (1-2)	25 (22.7)	1.4 (0.9)	1 (1-1)
Hiccups	63 (20.9)	2.8 (1.0)	3 (2-3)	24 (15.9)	1.3 (0.6)	1 (1-1)	19 (17.3)	1.3 (0.7)	1 (1-1)
Insomnia	173 (57.5)	3.1 (0.9)	3 (3-4)	71 (47)	1.9 (1.2)	1 (1-3)	49 (44.5)	1.8 (1.1)	1 (1-3)
Sleep disturbance	159 (52.8)	3.2 (0.9)	3 (3-4)	80 (53)	2 (1.2)	2 (1-3)	57 (51.8)	1.9 (1.1)	2 (1-3)
Lack of memory	115 (38.2)	2.7 (0.9)	2 (2-3)	53 (35.1)	1.6 (1)	1 (1-2)	37 (33.6)	1.6 (1)	1 (1-2)

Symptom is considered prevalent if ESAS score >2 or Likert score >1.

Abbreviations: ESAS: Edmonton Symptom Assessment Scale; SD: standard deviation.

Table 3 summarizes the SCs identified at baseline and again at D_15_ and D_30_, accounting for more than 40% of the total variance in symptom scores. As previously reported,^17^ 3 SCs were identified at baseline: NPM, gastro-intestinal (GI), and sleep impairment (SI) clusters. The constitution of some SCs, as initially identified, changed during follow-up: at baseline, the NPM cluster included tiredness, lack of appetite, dyspnea, depression, anxiety, and lack of well-being; on D_15_ an additional SC composed of pain, weight loss, dry mouth, and lack of memory was identified; on D_30_ while dyspnea and weight loss were no longer part of NPM, anxiety and dry mouth met the criteria for inclusion in the SI cluster. The GI cluster had 3 symptoms with a stable presence during the 3 assessments: nausea, vomiting, and hiccups, associated with dysphagia on D_15_. The SI cluster had insomnia and sleep disturbance as core symptoms at baseline and D_15_, and additionally, they were associated with anxiety and dry mouth on D_30_.

**Table 3.** Frequency and constitution of symptom clusters identified.

Symptom clusters														
Baseline (*n*=301)					Day 15 (*n*=150)					Day 30 (*n*=110)				
Symptoms	*N* (%) of patients	Factor loading score^*^	% of variance	Cronbach’s α	Symptoms	*N* (%) of patients	Factor loading score^*^	% of variance	Cronbach’s α	Symptoms	*N* (%) of patients	Factor loading score^*^	% of variance	Cronbach’s α
Neuro-psycho-metabolic cluster	131 (43.5)		17.31	0.736	Neuro-psycho-metabolic cluster	49 (32.5)		20.757	0.811	Neuro-psycho-metabolic cluster	40 (36.4)		16.843	0.762
Tiredness		0.718			Tiredness		0.657			Tiredness		0.626		
Lack of appetite		0.498			Lack of appetite		0.667			Lack of appetite		0.533		
Dyspnea		0.538			Dyspnea		0.481			Dyspnea		-		
Depression		0.684			Depression		0.635			Depression		0.534		
Anxiety		0.618			Anxiety		0.601			Anxiety		-		
Lack of well-being		0.703			Lack of well-being		0.675			Lack of well-being		0.577		
					Pain		0.504			Pain		0.626		
					Weight loss		0.574			Weight loss		-		
					Dry mouth		0.508			Dry mouth		-		
					Lack of memory		0.413			Lack of memory		0.515		
										Somnolence		0.620		
										Constipation		0.507		
Gastro-intestinal cluster	51 (16.9)		12.39	0.591	Gastro-intestinal cluster	22 (14.6)		14.059	0.728	Gastro-intestinal cluster	5 (4.5)		13.531	0.697
Nausea		0.600			Nausea		0.815			Nausea		0.851		
Vomiting		0.758			Vomiting		0.835			Vomiting		0.839		
Constipation		0.522			Constipation		-			Constipation		-		
Dry mouth		0.410			Dry mouth		-			Dry mouth		-		
Hiccups		0.545			Hiccups		0.552			Hiccups		0.418		
					Dysphagia		0.643			Dysphagia		-		
Sleep impairment cluster	139 (46.2)		9.99	0.864	Sleep impairment cluster	60 (39.7)		10.575	0.853	Sleep impairment cluster	47 (42.7)		12.131	0.665
Insomnia		0.869			Insomnia		0.824			Insomnia		0.750		
Sleep disturbance		0.828			Sleep disturbance		0.799			Sleep disturbance		0.684		
										Dry mouth		0.483		
										Anxiety		0.685		

Strikethrough means absence in the cluster of a symptom previously reported.

^*^Factor loading >0.40 was considered as an inclusion criteria.

At baseline,^6^ 105 patients (34.9%) had one SC, 79 (26.2%) had 2, and 18 (6.3%) had all SCs; on D_15_, 59 patients (39.1%) had one SC, 24 (15.9%) had 2 SC, and 8 (5.3%) had all 3 SC. On D_30_, 41 (37.3%) had one SC, 24 (21.8%) had 2 SC, and one (9%) had all 3 SCs. Regarding D_15_ and D_30_, the NPM, GI, and SI clusters demonstrated internal consistency with good Cronbach’s alpha coefficient values (D_15_: 0.81, 0.73, and 0.85, respectively and D_30_: 0.76, 0.70, and 0.67, respectively).

**Figure 2.**
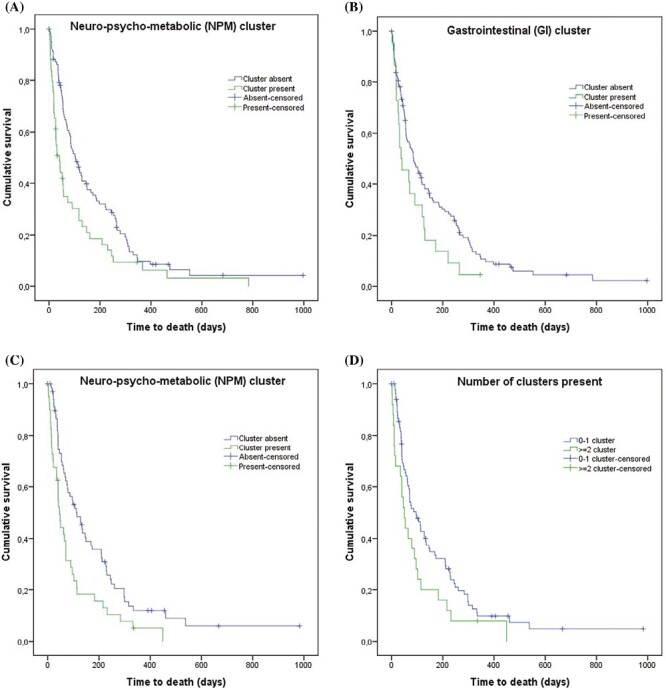
Kaplan-Meier survival estimates according to the presence of symptom cluster. (**A**) Neuro-psycho-metabolic (NPM) cluster at day 15 (*D*_15_). (**B**) Gastrointestinal (GI) cluster at day 15 (*D*_15_). (**C**) Neuro-psycho-metabolic (NPM) cluster at day 30 (*D*_30_). (**D**) Number of symptom clusters present at day 30 (*D*_30_).

The strength of association of the SCs with survival is summarized in Table 4. The median survival of the entire study cohort with D_0_ as baseline was 37 (95%CI, 28-46) days, whereas median survival from D_15_ was 85 (95%CI, 60-110) days, and from D_30_, it was 75 (95%CI, 49-101) days. A statistically significant reduction in survival was observed for patients with the NPM cluster (baseline: 23 vs. 58 days, *P* < .001; D_15_: 41 vs. 104 days, *P* =.004; D_30_: 46 vs. 114 days, *P* = .002, Fig. 2A and 2C). A reduced survival was also observed for patients included in the GI cluster on D_15_ and D_30_ but not on baseline (D_15_: 37 vs. 86 days, *P* = .046; D_30_: 53 vs. 77 days, *P* = .040, Fig. 2B). No survival differences were found for patients included in SI cluster at the 3 evaluation time points.

**Table 4.** Association of the SCs with survival.

	Cluster present		Cluster absent		*P*
	*n*	Survival median (95%CI)	*n*	Survival median (95%CI)	
NPM cluster					
Baseline	131	23 (17-29)	170	58 (41-75)	<.001
*D*_15_	49	41 (22-60)	102	104 (74-134)	.004
*D*_30_	40	46 (33-59)	70	114 (68-160)	.002
NGI cluster					
Baseline	51	29 (9-49)	250	39 (27-51)	.194
*D*_15_	22	37 (1-72)	129	86 (59-113)	.046
*D*_30_	60	53 (0-111)	105	77 (13-50)	.040
SI cluster					
Baseline	139	30 (18-42)	162	41 (27-55)	.884
*D*_15_	60	85 (41-129)	91	78 (52-104)	.828
*D*_30_	47	77 (51-103)	63	75 (21-129)	.222

*P*-values were obtained by log-rank test.

Abbreviations: CI: confidence interval; D_15_: day 15: D_30_: day 30; NPM: neuro-psycho-metabolic; GI: gastro-intestinal; SI: sleep impairment.

Considering the number of SCs for any given patient, the presence of 2 or more SCs at baseline and D_30_ was associated with survival (*P* = .005 and *P* = .040, respectively), with median survival estimates 21 (95%CI, 17-25) and 50 (95%CI, 28-71) days for D_0_ and D_30_, respectively. In contrast, among those who had one or none of the 3 clusters, the median survival estimate was 45 (95%CI, 32-58) days for D_0_, and 96 (95%CI, 62-130) for D_30_ (Fig. 2D).

In the univariable Cox regression analysis, variables potentially influencing time to death at 30 days, with a *P* < .25, were selected for the multivariable model, summarized on Table 5. Thirty days after D_15_, hospitalization at inclusion (HR: 2.43, 95%CI, 1.18-5.01; *P* = .016), poor PS (HR: 2.80, 95%CI, 1.03-7.58; *P* = .043), and NPM cluster presence (HR: 2.51, 95%CI, 1.25-5.05; *P* = .009) were independently associated with worse survival. Regarding the evaluation at D_30_, the hospitalization at inclusion (HR: 3.41, 95%CI, 1.35-8.62; *P* = .009) and NPM cluster presence (HR: 3.90, 95%CI, 1.54-9.86; *P* = .004) remained significantly associated with time to death after 30 days of follow-up, but also GI cluster presence (HR: 5.71, 95%CI, 1.20-27.26; *P* = .029) was associated with worse survival.

**Table 5.** Uni- and multivariable cox regression analysis.

	Association with survival at 30 days								
	Baseline			D_15_			D_30_		
	Univariable analysis	Multivariable analysis		Univariable analysis	Multivariable analysis		Univariable analysis	Multivariable analysis	
	*P*	HR (95%CI)	*P*	*P*	HR (95%CI)	*P*	*P*	HR (95%CI)	*P*
Demographics									
Age	<.001	0.98 (0.97-0.99)	.008	023	-	-	.054	-	-
Sex	.417	-	-	.981	-	-	.870	-	-
Hospital status	<.001	2.27 (1.47-3.51)	<.001	<.001	2.43 (1.18-5.01)	.016	.013	3.41(1.35-8.62)	.009
ECOG PS	<.001	1.90 (1.24-2.89)	.003	.001	2.80 (1.03-7.58)	.043	.173	-	-
Metastatic site									
Lymph nodes	.123	-	-	.189	-	-	.461	-	-
Lung	.214	-	-	.613	-	-	.358	-	-
Liver	.197	-	-	.539	-	-	.240	-	-
Pelvic	.781	-	-	.511	-	-	.800	-	-
Peritoneal	.523	-	-	.334	-	-	.741	-	-
Bone	.700	-	-	.839	-	-	.187	-	-
CNS	.592	-	-	.421	-	-	.353	-	-
Symptom cluster									
NPM cluster	<.001	1.64 (1.17-2.31)	.005	<.001	2.51 (1.25-5.05)	.009	.004	3.90 (1.54-9.86)	.004
GI cluster	.260	-	-	.182	-	-	.136	5.71 (1.20-27.26)	.029
SI cluster	.098	-	-	.295	-	-	.849	-	-
Number of clusters (≥2)	<.001	-	-	.019	-	-	.024	-	-

*P*-values were obtained by Cox regression models.

Abbreviations: CI: confidence interval; CNS, central nervous system; D_15_, day 15; D_30_, day 30; ECOG: Eastern Cooperative Oncology Group scale; GI: gastro-intestinal; HR: hazard-ratio estimate; NPM: neuro-psycho-metabolic; PS: functional performance status; SI: sleep impairment.

## Discussion

Our prospective analysis showed that symptoms’ prevalence and median intensity scores decreased over time. In addition, among all 3 SCs identified, SI was the most frequent SC and showed longitudinal stability of symptoms, encompassing insomnia and sleep disturbance throughout the study. By contrast, the NPM cluster had constitutional variation across the 3 time points but still maintained an association with shorter survival over time.

After inclusion in the palliative care program, our data showed a reduction in prevalence and median intensity scores of all individual symptoms evaluated from baseline to D_30_. The most common symptoms reported at baseline were tiredness and pain, consistent with previous studies.^24,25^ Pain prevalence at baseline as well as the observation of pain improvement over the first month were in line to prior reports.^26,27^ Other studies have reported longer time to pain control especially in cases of neuropathic pain component, female sex, and baseline use of adjuvant analgesics.^28^ However, the biopsychosocial complexity of cancer pain and the potential cultural differences may limit definitive conclusions in this regard.^29^

Major symptom intensity variation was also noticed with nausea and dyspnea, also observed in previous reports.^30^ By the contrary, a significant decrease in dyspnea over time was not expected, as previous published data showed that dyspnea typically increases in prevalence and intensity as patients approach the last weeks of life.^31-33^ However, given the fact that most of our patients were hospitalized at time of inclusion, multimodal interventions delivered by interprofessional teams in this setting may have been beneficial in controlling this symptom.

Our main objective was to report on SC stability during disease progression and determine their strength of association with survival in patients with advanced cancer. In this study, all symptoms evaluated were included in at least 1 of the 3 SCs identified, although their constitution had some changes between the baseline, D_15_, and D_30_ assessments.

The most frequently identified SC in the 3 evaluation periods was SI (range: 39.7%-46.2%). Although data on sleep disturbance in patients with advanced cancer are limited, patients in palliative care were reported to have a prevalence of 60.8% for consistent sleep disturbances over a 6-month period.^34^ Meanwhile, sleep disorder disturbance has been reported as being strongly correlated with anxiety and depression.^35^

NPM was the second most common SC over the study period (32.5% to 43.5%). This SC had a constitutional variation over time. The greatest change occurred at D_30_ where dyspnea, anxiety, weight loss, and dry mouth were no longer part of the cluster; instead, the association at this point was with drowsiness and constipation. Despite this variation, depression, tiredness, lack of well-being, and lack of appetite represented the consistent core symptoms, suggesting an interrelationship between those symptoms within the NPM cluster. Co-occurring anxiety and depression symptoms within a neuropsychological cluster have been identified in multiple studies in patients with advanced cancer,^11^ including with brain^36^ and bone^37^ metastases. One study found longitudinal stability of the anxiety-depression cluster, while symptoms’ severity decreased suggesting significant improvement after initial palliative care expert consultation.^38^

The GI was the least frequent cluster with a reduction in its prevalence over time (D_0_: 16.9%; D_30_: 4.5%), also presenting the lowest internal consistency amongst the identified SCs. Its frequency was lower than that described in other studies with patients with advanced cancer, probably because our study sample had a lower proportion of patients with gastrointestinal tumors and none under active antineoplastic therapy when compared to previously published data.^39,40^

To date, longitudinal stability of SCs in patients with advanced cancer receiving best supportive care is unknown, as very few studies have addressed this question.^11,40^ As a dynamic construct, longitudinal investigation of SCs still presents considerable methodological unresolved issues.^9-11^ In 2007, Kirkova and Walsh^41,42^ suggested a method to evaluate SCs’ stability that required 75% of the symptoms in a cluster to be present across different timepoints or dimensions. Alternatively, other studies have defined stability in terms of a reproducibility of a specific cluster detected throughout time.^43^ In 2016, Barsevick^6^ defined stability as to how clusters emerged consistently across statistical approaches, similar populations, or over time. To date, the optimal statistical “cutoff” points to define SCs and the optimal timing of longitudinal evaluation have not been established.

Taking into consideration the scarcity of data on longitudinal assessment of SCs in patients with advanced cancer, most studies included in a systematic review found that SCs did not display longitudinal stability over time.^11^ However, most studies were performed in patients undergoing palliative radiotherapy, and longitudinal stability was assessed over a postradiation period using a preradiation time point as baseline.^44,45^ These findings suggest that the effect of radiation on pain relief may influence other symptoms within the SCs.

We have previously reported a prognostic value in relation to survival of NPM cluster and presence of 2 or more SCs.^17^ Interestingly, in the longitudinal analysis of the current study, the presence of the NPM cluster maintained an association with shorter survival over time. Unlike the baseline assessment, the presence of the GI cluster in subsequent evaluations at D_15_ and D_30_ was also associated with a significantly shorter median survival. Meanwhile, our findings suggest that the presence of the SI cluster had no prognostic significance.

Our data are consistent with previous studies that found a negative association between SCs and prognosis in patients with advanced cancer.^15,16^ However, the limited and heterogeneous sample size, as well as different methods in SCs’ definition, makes it difficult to draw definite conclusions.

Aktas et al^16^ identified a fatigue/anorexia-cachexia cluster with a strongest statistically significant negative impact on survival. The symptoms within this SCs are similar to our NPM cluster, with the exception of anxiety and depression.^16^ Using a quality-of-life questionnaire as a symptom assessment tool, Koyama et al^46^ identified a similar cluster that included dyspnea, appetite loss, fatigue, and nausea, as a significant prognostic indicator in terminally ill patients with advanced cancer. The presence of this SC provided an approximately 2-fold increased risk of mortality.^46^

Furthermore, the presence of more than one SCs was associated with reduced median overall survival at baseline and at 4 weeks, suggesting that patients with advanced cancer survival decreases with increasing concomitant SCs, which is also consistent with previously published data.^15,16^ Our results support the clinical relevance of examining survival as an outcome of SCs’ research.

Limitations of this study include an heterogeneous population of patients with advanced cancer with different primary tumors in a single institution that may not be representative of cancer population. In addition, almost two thirds of the patients were hospitalized at baseline, which may reflect complications associated with advanced cancer that may have influenced the results and impact on prognosis. Second, because we excluded patients with cognitive impairment, this could result in further selection bias and inaccurate characterization of actual SCs for patients with advanced cancer receiving best supportive care. The use of short questionnaires to assess the frequency and intensity of symptoms may have influenced the SCs identified. The addition of 10 additional symptoms to the ESAS-r scale was our attempt to mitigate this possible limitation. Finally, we acknowledge the long time between data collection and data analysis and publication of the results which can be justified by a numbers of factors: the PI of this study (PB) relocated from the Institution where this project took place (Lisbon, Portugal) to a different Institution in the US (Cleveland, OH). The access to the data was initially restricted to the PI, co-PI, and statistical team; the PI was then able to transfer the ownership of the project to the new PI (DS) and coordinate the data analysis with the statistical team (ALP, MA) and prepare the current manuscript. Nonetheless, the authors believe that this data remains clinically relevant in this area of supportive oncology and end of life care.

### Relevance for Medical Practice and Clinical Research

Given that SCs may have a prognostic value, the importance of regular assessment of symptom burden and SCs in clinical practice should not be underestimated. Despite the need for prospective studies to evaluate the influence of SCs on therapeutic interventions, identification of SCs could provide useful information to guide clinical decisions. However, there is a strong need for agreement in SCs definitions and reliable assessment methods, as well as their optimal timing. Furthermore, future research is needed to determine the associations between SCs and patient-reported outcomes.

## Conclusions

In this prospective study, we identified 3 clinically relevant SCs in patients with advanced cancer, with different temporal stabilities. The constitution of the SCs had small variations over time, resulting in the maintenance of a stable set of nuclear symptoms. The presence of a NPM cluster and the occurrence of hospitalization maintained their prognostic value over time. Further studies are needed to investigate the efficacy of potential therapeutic interventions on these patient groups.

## Data Availability

The data underlying this article will be shared on reasonable request to the corresponding author.
